# Antibacterial and Cytotoxic Bridged and Ring Cleavage Angucyclinones From a Marine *Streptomyces* sp

**DOI:** 10.3389/fchem.2020.00586

**Published:** 2020-08-04

**Authors:** Lin Guo, Lu Zhang, Qiaoli Yang, Bo Xu, Xinzhen Fu, Ming Liu, Zhi Li, Shumin Zhang, Zeping Xie

**Affiliations:** ^1^School of Pharmacy, Binzhou Medical University, Yantai, China; ^2^College of Life Sciences, Yantai University, Yantai, China

**Keywords:** angucyclinones, oxygen bridge, ring cleavage, structure elucidation, *Streptomyces pratensis*, antibacterial activity, cytotoxicity

## Abstract

Chemical investigation of a marine-derived *Streptomyces* sp. KCB-132, cultivated in liquid ISP2 medium, had led to the discovery of three C-ring cleavage angucyclinone *N*-heterocycles, pratensilins A–C, with a novel spiro indolinone-naphthofuran skeleton. Addition of 50 μM LaCl_3_ to the same medium and subsequent chemical analysis of this strain returned a new member of this rare class, pratensilin D (**1**), along with two new angucyclinone derivatives, featuring ether-bridged (**2**) and A-ring cleavage (**3**) structural properties. Their structures and absolute configurations were assigned by spectroscopic analysis, single-crystal X-ray diffractions, and equivalent circulating density (ECD) calculations. (+)- and (–)-**1**, a pair of enantiomeric nitrogen-containing angucyclinones, exhibited different strengths of antibacterial and cytotoxic activities.

## Introduction

Angucyclines and angucyclinones (sugarless) represent the largest family of type II polyketide synthase (PKS)-engineered natural products (Rohr and Thiericke, 1992; Krohn and Rohr, 1997; Kharel et al., 2012), which share a characteristic tetracyclic benz[*a*]anthracene core and exhibit a wide range of biological activities (Shaaban et al., 2012; Ma et al., 2015; Xie et al., 2016; Yixizhuoma et al., 2017; Liu et al., 2019; Wu et al., 2019). Actinobacteria are the exclusive producers of angucyclines and angucyclinones that have been proven to be a prolific source of antibiotics for the production of 45% of all reported microbial active metabolites (Bérdy, 2005; Arens et al., 2013). However, only a few such metabolites are detected in the laboratory due to conditional or low production of most metabolites biosynthesized by cryptic gene clusters (Helge et al., 2002; Demain and Sanchez, 2009; Newman and Cragg, 2012; Bethany and Mohammad, 2017). Rare earth, such as scandium, was reported to be an effective factor that induces or stimulates the production of secondary metabolites (Kawai et al., 2007).

**Figure 1:**
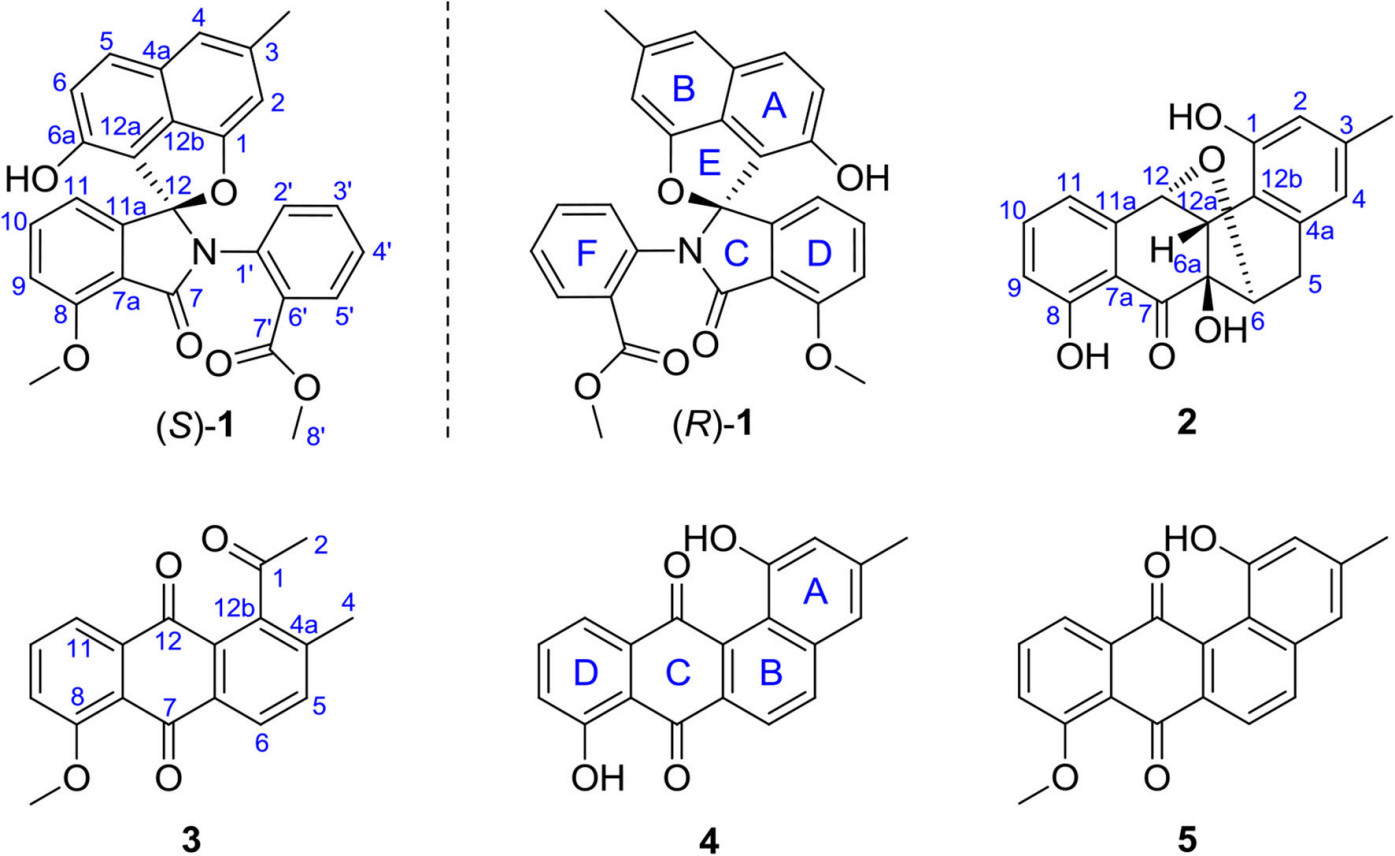
Structures of the isolated angucyclinones.

Our previous chemical analysis of the metabolites from marine actinomycete *Streptomyces pratensis* KCB-132 have led to the discovery of three pairs of unprecedented ring C-modified angucyclinone *N*-heterocycles, (+)- and (–)-pratensilins A–C. Addition of the rare earth, lanthanum, to the liquid ISP2 medium and repeated purification of the organic extract of this strain resulted in the isolation of two new ring cleavage angucyclinones, named (±)-pratensilin D (**1**) and pratensinon A (**3**), and a new ether-bridged analog, kiamycin E (**2**), together with two known angucyclinones tetrangulol (**4**) and 8-*O*-methyltetrangulol (**5**) (Figure 1). Herein, we report the isolation, structure characterization, and biological activity of compounds **1**–**3**, and a plausible biogenetic pathway for **1** and **2** is also proposed.

## Results and Discussion

**Figure 2:**
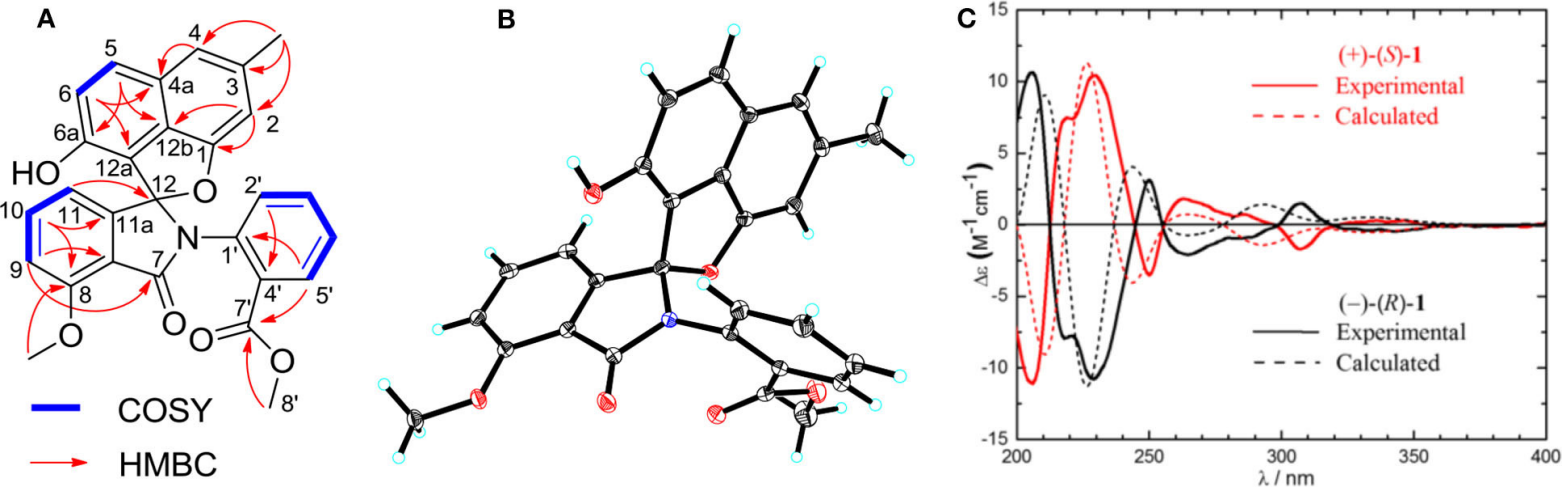
**(A)** Key ^1^H-^1^H COrrelation SpectroscopY (COSY) and heteronuclear multiple bond correlations (HMBCs) of **1**. **(B)** X-ray crystal structure of (–)-**1**. **(C)** Experimental and calculated equivalent circulating density (ECD) spectra for (+)-(*S*)-**1** and (–)-(*R*)-**1**.

Compound **1** was obtained as a colorless solid. Its molecular formula of C_28_H_21_O_6_N was established from the positive-ion HRESIMS peak at *m/z* 468.14418 [M + H]^+^ (calcd for C_28_H_22_O_6_N, 468.14471). The ^1^H, ^13^C, and heteronuclear single quantum coherence (HSQC) NMR spectra of **1** displayed one tertiary methyl at δ 2.35, two methoxy groups at δ 3.75 and 3.97, and 11 aromatic methines (δ 7.61, 7.55, 7.54, 7.39, 7.31, 7.25, 7.15, 7.08, 6.96, 6.60, and 6.41), as well as one exchangeable proton at δ 10.11. The ^13^C NMR spectrum indicated the presence of two carbonyls at δ 165.1 and 166.4 and 12 quaternary carbons between δ 103.7 and 157.2 (Table 1). Interpretation of the ^1^H-^1^H COrrelation SpectroscopY (COSY) spectrum permitted three isolated fragments to be established. The first fragment, a 1,2,3-trisubstituted benzene ring (ring D) was assigned by ^1^H-^1^H COSY cross-peaks for H-9/H-10/H-11 along with heteronuclear multiple bond correlations (HMBCs) from H-9 to C-7a and from H-10 to C-8 and C-11a. Next, ^1^H-^1^H COSY cross-peaks for H-2′/H-3′/H-4′ and H-5′ along with HMBC couplings from H-2′ to C-6′ and from H-5′ to C-1′ allowed the constitution of a second benzene ring (ring F). The last fragment was established based on ^1^H-^1^H COSY cross-signals between H-5 and H-6 in combination with HMBCs from H-5 to C-6a and C-12b and from H-6 to C-4a and C-12a, resulting in a third benzene ring as ring B, which shared double bond Δ^4a,12b^ with a methyl benzene moiety (ring A) to fuse into a naphthalene system, as indicated by HMBCs from H-2 to C-1 and C-12b, from 3-CH_3_ to C-2, C-3, and C-4, and from H-4 to C-4a (Figure 2A).

**Table 1:** ^1^H (600 MHz) and ^13^C (150 MHz) NMR Data of Compounds **1**–**3**.

**No**.	**1**^**a**^		**2**^**a**^		**3**^**b**^	
	**δ_**C**_**	**δ_**H**_ (*J* in Hz)**	**δ_**C**_**	**δ_**H**_ (*J* in Hz)**	**δ_**C**_**	**δ_**H**_ (*J* in Hz)**
1	156.4		154.3		205.7	
2	103.7	6.41, s	113.4	6.48, s	31.1	2.59, s
3	136.0		137.0			
4	115.7	6.96, d (1.0)	120.4	6.45, s	19.0	2.38, s
4a	125.1		135.9		139.1	
5	127.8	7.54, d (8.6)	35.3	3.10, d (16.9) 2.82, dd (16.9, 2.8)	136.6	7.63, d (7.9)
6	121.4	7.08, d (8.6)	74.8	4.06, t (2.8)	127.7	8.22, d (7.9)
6a	149.3		81.9		133.4	
7	165.1		205.8		181.8	
7a	116.5		114.8		121.0	
8	157.2		162.2		160.4	
9	114.0	7.25, d (7.9)	117.6	6.97, d (7.9)	118.3	7.36, dd (8.5, 1.1)
10	135.6	7.55, t (7.9)	137.4	7.56, t (7.9)	135.2	7.73, dd (8.5, 7.7)
11	115.0	6.60, dd (7.9, 0.5)	117.2	6.98, d (7.9)	120.1	7.89, d (7.7, 1.1)
11a	146.4		148.8		135.3	
12	104.6		85.0	4.86, s	183.8	
12a	114.8		49.0	3.65, s	129.1	
12b	128.2		120.9		142.6	
1'	133.8					
2'	131.2	7.15, dd (7.7, 1.4)				
3'	132.5	7.39, td (7.7, 1.6)				
4'	129.1	7.31, td (7.7, 1.4)				
5'	130.4	7.61, dd (7.7, 1.6)				
6'	132.9					
7'	166.4					
8'	52.6					
3-CH_3_	22.7	2.35, s	21.4	2.18, s		
8-OCH_3_	56.3	3.92, s			56.6	4.06, s
1-OH		10.29, s		9.30, s		
6a-OH		10.11, s		6.08, s		
8-OH				11.69, s		

a Recorded in DMSO-d_6_,

b *Recorded in CDCl_3_*.

In addition, key HMBCs from H-9 to the carbonyl carbon C-7 (δ 165.1) and from H-11 to the deshielded carbon C-12 (δ 104.6) extended ring D to C-7 and C-12; these two carbons had to be connected by an oxygen or a nitrogen atom to form either an isobenzofuran-1-one or an isoindolin-1-one ring system in line with both the chemical shifts and the molecular composition. However, both ring systems had appeared in the structures of C-ring cleavage angucyclinones produced by the same strain, previously. Moreover, no obvious correlations were observed between ring F and other ring systems in the ^1^H-^1^H COSY and HMBC spectra, which made it problematic to assign the complete structure by spectroscopic analysis.

Finally, the structure of **1** was elucidated by X-ray crystallographic analysis (Figure 2B). Slow evaporation of a MeOH/CH_2_Cl_2_ solution of **1** provided suitable crystals. X-ray experiment revealed that **1** was composed of a methyl benzoate moiety attached to a spiro indolinone-naphthofuran core skeleton. Additionally, the X-ray data exhibited a centrosymmetric space group *C*12/*c*1, supporting a racemic nature. Subsequent optical resolution of **1** was achieved by high-performance liquid chromatography (HPLC) equipped with a chiral column to give (+)-**1** and (–)-**1**. Based on the analogy to pratensilins A–C, we suggest that the absolute configurations for the two isomers can be assigned as (+)-*S*-**1** and (–)-*R*-**1** for a biogenetic reasoning. As in pratensilins A–C, the (*S*)-enantiomers have positive optical rotations, while the (*R*)-enantiomers share consistently negative optical rotations (Zhang et al., 2017). This assignment was also confirmed by equivalent circulating density (ECD) calculations using the same ECD computational approach as for pratensilins A–C. In fact, the CD spectra of (+)-**1** and (–)-**1** reproduced well with those of pratensilins A–C apart from a systematic wavelength shift (Figure 2C). Herein, we suggest to name pratensilin D for compound **1**.

**Figure 3:**
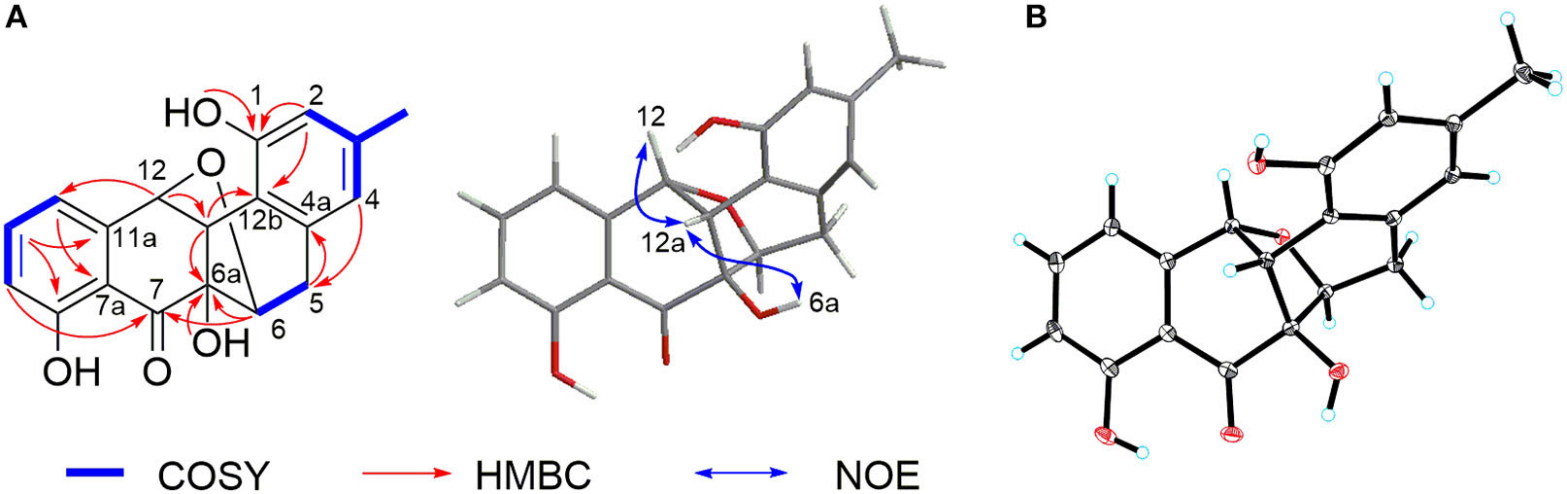
**(A)** Key ^1^H-^1^H COrrelation SpectroscopY (COSY), heteronuclear multiple bond correlation (HMBC), and nuclear Overhauser effect (NOE) correlations of **2**. **(B)** X-ray crystal structure of **2**.

Compound **2** was isolated as a pale-yellow solid. High-resolution electrospray ionization mass spectrometry (HRESIMS) provided a molecular formula of C_19_H_16_O_6_ [*m/z* 325.10740 [M + H]^+^, calcd for C_19_H_17_O_5_, 325.10760] requiring 12 degrees of unsaturation. The ^13^C and distortionless enhancement by polarization transfer (DEPT) NMR spectra exhibited 19 carbon signals for one methyl, one methylene, eight methines including five aromatic methines, one carbonyl carbon, and eight quaternary carbons, of which seven are sp^2^ aromatic carbons (Table 1). Since the aromatic carbons and the carbonyl accounted for 7 of the 12 degrees of unsaturation, compound **2** must be pentacyclic. In the ^1^H NMR spectrum, the characteristic signals for *o*-coupled protons at δ 6.97 (H-9), δ 7.56 (H-10), and δ 6.98 (H-11) indicated the presence of a 1, 2, 3-trisubstituted benzene ring D, while a typical olefinic methyl resonance at δ 2.18 (3-CH_3_), which showed long-range couplings with two aromatic singlets at δ 6.48 (H-2) and δ 6.45 (H-4), was assigned to benzene ring A as observed in **1** (Figure 1). An additional ^1^H-^1^H COSY cross-peak between H_2_-5 and H-6 along with HMBCs from H_2_-5 to C-4a, from H-6 to C-6a, and from H-12a to C-6a and C-12b suggested that the cyclohexene ring B is joined to ring A by sharing the double bond between C-4a and C-12b. Furthermore, ring systems A/B and D were linked by ring C (C-7 and C-12) to form an angucyclinone skeleton evidenced by HMBCs from both H-6 and H-9 to the carbonyl carbon C-7 (δ 205.8) and from H-12 to C-11 and C-12a (Figure 3A).

Positioning of two hydroxyl groups at C-1 (δ 154.3) and C-6a (δ 81.9) was facilitated by HMBCs from 1-OH to C-1 and from 6a-OH to C-6a, respectively. The remaining phenolic OH group at δ 11.69 had to be attached to the oxygenated aromatic carbon C-8 to fit in the deshielded characteristic of C-8 (δ 162.2). At this point of the structure elucidation, only one oxygen atom was left as defined by the molecular formula, the two downfield methine carbons C-6 and C-12 must be joined by an oxygen bridge in agreement with both the chemical shifts of C-6 (δ 74.8) and C-12 (δ 85.0), completing the pentacyclic requirement of **2**, which was further supported by three-bond HMBCs coupling from H-6 to C-12 (and *vice versa*). Taken together, a rare 6,12-epoxybenz[*a*] anthracene ring topology was expected; we thus suggest the successive name kiamycin E for **2**.

The relative configuration of **2** was tentatively assigned by nuclear Overhauser effect (NOE) correlations (Figure 3A). The NOE contacts of H-12 with H-12a and of H-12a with 6a-OH indicated that H-12, H-12a, and 6-OH have common β-orientations, which require an α-orientation of the bridged C-6/12 bond; consequently, H-6 was defined as β, although no NOE was observed between H-6 and 6a-OH. This assignment was finally confirmed by single-crystal X-ray diffraction experiment (Figure 3B). Additionally, the absolute configuration was assigned as 6*S*, 6a*R*, 12*R*, and 12a*S*, respectively.

**Figure 4:**
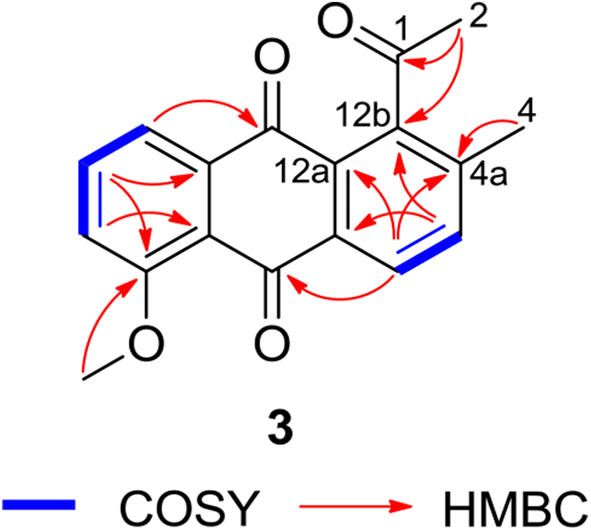
Key ^1^H-^1^H COrrelation SpectroscopY (COSY) and heteronuclear multiple bond correlations (HMBCs) of **3**.

Compound **3** was isolated as a yellow powder. Its molecular formula was assigned to be C_18_H_14_O_4_ based on HRESIMS [*m/z* 295.09677 [M + H]^+^, calcd for C_18_H_15_O_4_, 295.09703]. In the ^13^C NMR spectrum, the typical carbonyl carbon resonances at δ 181.8 and 183.8 indicated the presence of an anthraquinone skeleton. A comparison of the ^1^H and ^13^C NMR spectra of **3** and the known derivative **4** revealed superimposable resonances for the anthraquinone moiety, apart from ring A. Resonances at δ 31.1 and 205.7 indicated the presence of an acetyl group, which was positioned at C-12b of ring B by HMBCs from 2-CH_3_ to the carbonyl carbon C-1 and the quaternary carbon C-12b (δ 142.6). An additional HMBC from 4-CH_3_ (δ 2.38) to C-4a (δ 139.1) permitted the methyl group to be located at C-4a (Figure 4). At this point, all carbon and proton resonances of **3** were assigned, leading to a structure feature of A-ring cleavage, which is very rare in the angucyclinone/angucycline family. For better comparison, compound **3** was trivially named pratensinon A, and the conventional angucyclinone numbering was used for compounds **1**–**3** in this paper.

**Scheme 1:**
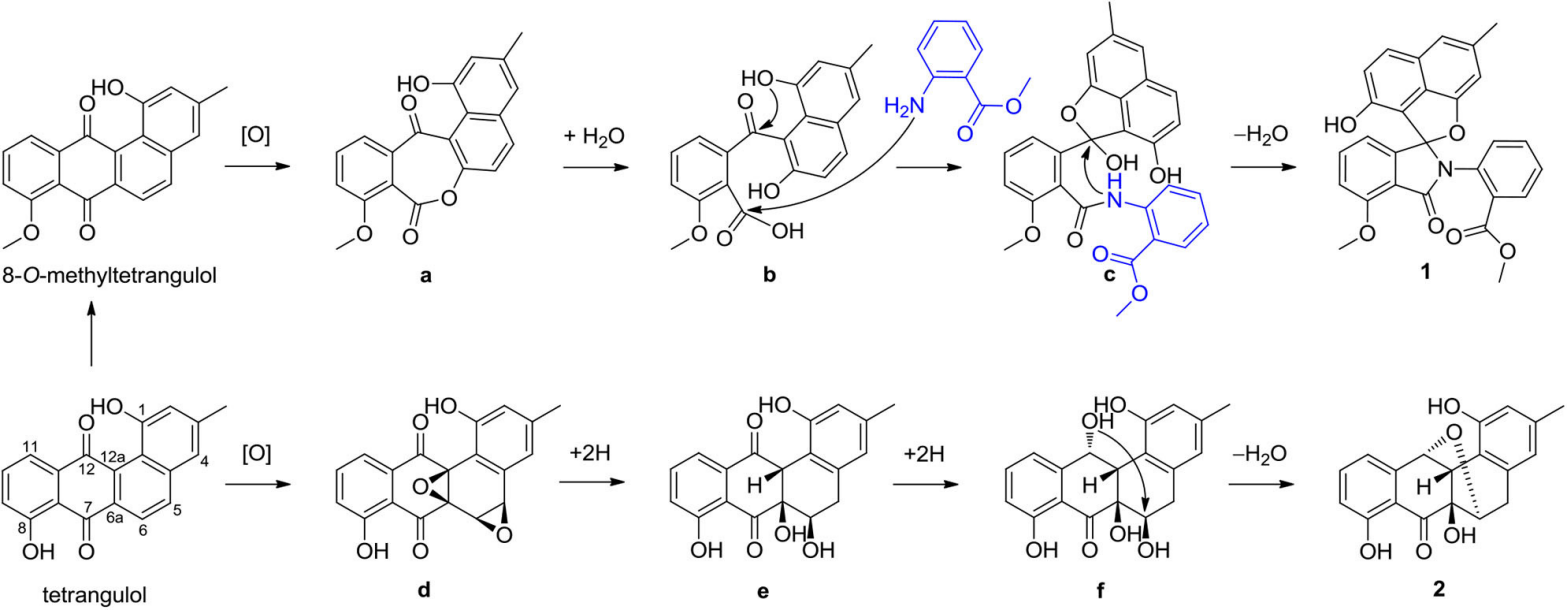
Plausible biosynthetic pathway of compounds **1** and **2**.

Compound **1** represents a C-ring cleavage angucyclinone with a rare nitrogen-containing spiro ring system that is distinct from the bridged counterpart **2**. However, these two compounds might originate from the same angucyclinone precursor tetrangulol (**4**), which together with 8-*O*-methyltetrangulol (**5**), were isolated from the same strain in parallel, and a plausible biosynthetic pathway is proposed in Scheme 1. Starting with methylation on **4** results in ester **5**, which on Baeyer–Villiger oxidation gives lactone **a**, and continues through hydrolytic cleavage to form intermediate **b**, followed by acetalization and condensation at C-7 ketone by 2-(methoxycarbonyl) aniline to introduce a nitrogen atom in **c**, which can be intramolecularly cyclized into pratensilin D. In another pathway, oxidation on the double bond Δ^5,6^ and Δ^6a,12a^ furnishes epoxide **d**, which undergoes a series of reduction to yield intermediate **f**, and subsequent cyclization gives rise to kiamycin E.

Angucyclines and angucyclinones possess not only great structural diversity but also a broad range of biological activities, predominantly anticancer and antibacterial activities (Kharel et al., 2012). Compounds **1**–**3** were thus screened for activity against Gram-positive (*Bacillus cereus* CMCC 32210, *Staphylococcus aureus* CMCC 26003) bacteria and yeast (*Candida albicans* CMCC 98001), as well as five human cancer cell lines (NCI-H460, PANC-1, Colon 38, Hela, and HepG2) (Table 2). (–)-**1** exhibited selective inhibitory activity against *B. cereus* CMCC 32210 with a minimum inhibitory concentration (MIC) value of 4 μg/ml; in contrast, its enantiomer (+)-**1** showed no efficacy against all tested strains up to 64 μg/ml, so did **2** and **3**. Furthermore, (–)-**1** exhibited moderate cytotoxicity to NCI-H460 and HepG2 cell lines with respective IC_50_ values of 4.6 and 9.3 μg/ml, while (+)-**1** was only less active to NCI-H460 cells with an IC_50_ value of 9.2 μg/ml. **3** displayed cytotoxic activity to Colon 38 and Hela cells, with IC_50_ values of 7.3 and 10.3 μg/ml, respectively. However, **2** showed no inhibitory effect against all tested cell lines in the testing concentration range (IC_50_ >50 μg/ml).

**Table 2:** Cytotoxic and antimicrobial activities of compounds (+)- and (−)**-1**, **2**, and **3**.

**Compounds**	**Cytotoxic activity (IC**_****50****_ **μg/ml)**					**Antimicrobial activity (MIC μg/ml)**		
	**NCI-H460**	**PANC-1**	**HepG2**	**Colon38**	**Hela**	***C. albicans***	***S. aureus***	***B. cereus***
**(+)-1**	9.4	NT	>50	NT	NT	>64	>64	>64
**(–)-1**	4.9	NT	9.3	NT	NT	>64	>64	4
**2**	NT	NT	>50	>50	>50	>64	>64	>64
**3**	NT	NT	>50	7.3	10.3	>64	>64	>64
Nystatin	NT	NT	NT	NT	NT	4	NT	NT
Penicillin	NT	NT	NT	NT	NT	NT	4	2
Adriamycin	0.03	0.27	0.81	0.20	0.02	NT	NT	NT

*MIC, minimum inhibitory concentration; NT, not tested*.

In summary, five angucyclinones, including three new compounds (**1**–**3**) were isolated from the ISP2 medium fermentation with addition of 50 μM LaCl_3_ of the marine-derived *Streptomyces pratensis* strain KCB-132. The new compounds possessed different types of structural properties including oxygen bridge (**2**) and C-ring and A-ring cleavage (**1** and **3**), respectively. (+)- and (–)-pratensilin D (**1**), a pair of enantiomeric angucyclinone *N*-heterocycles, were further separated by chiral HPLC and showed different strengths of biological activities. The new structural properties, especially A-ring and C-ring cleavage disrupting the characteristic tetracyclic ring frame, expand the structural diversity of angucyclines and angucyclinones and provide insight into their structure–activity relationship.

## Experimental Section

### General Experimental Procedures

Opitical rotations were measured on an Autopol VI (Serial #91058) manufactured by Rudolph Research Analytical, Hackettstown, NJ, USA. UV spectra were recorded by a Shimadzu UV-2401PC spectrometer. CD spectra were recorded using a JASCO J-810 spectropolarimeter. NMR spectra were recorded on Bruker Avance III 600 spectrometers. Electrospray ionization (ESI)-high-resolution mass spectrometry (HRMS) were recorded on an Agilent G6230 TOF spectrometer. Single crystal X-ray crystallography was determined on SMART APEX II DUO X-ray single crystal diffractometer using Cu Kα radiation. Preparative HPLC was performed on a Waters 2489 series instrument with a UV/Visible detector, using a reversed-phase C18 column (Phenomenex, 250 mm × 21.2 mm, 5 μm). Chiral HPLC was carried out on an Agilent 1260 liquid chromatograph, utilizing chiral analytical columns [(*R, R*) WHELK-01 column, 4.6 mm × 250 mm, 10 μm, 100 A].

### Cultivation and Culture Extraction

*Streptomyces* sp. strain KCB-132 was isolated from a sediment sample collected off Kiaochow Bay, China, as described previously (Zhang et al., 2017). The sequence is deposited in GenBank under accession no. KX033803. The strain KCB-132 was cultured in seawater-based ISP2 medium (5 g of malt extract, 4 g of yeast extract, 4 g of glucose, 500 ml of deionized water, and 500 ml of seawater, pH 7.8) with the addition of 50 μM lanthanum chloride, at a total volume of 21.6 L (72 × 0.3 L), for 10 days at 28°C. The culture broth was filtered to provide filtrate and mycelium. The filtrate was absorbed onto XAD-16 amberlite resin, and the resin was eluted with methanol, then dried out methanol under reduced pressure. The resulting aqueous layer was extracted with ethyl acetate, while the mycelium was extracted by ethyl acetate under ultrasonic radiation directly; both ethyl acetate phases were combined to yield 7.2 g crude extract.

### Isolation of Compounds 1–5

The extract (7.2 g) was fractioned by silica gel column chromatography (40 g) and eluted with a step gradient of CH_2_Cl_2_ and MeOH. The CH_2_Cl_2_/MeOH 99:1 fraction was purified by preparative HPLC (Gemini, C18, 21.2 mm × 250 mm, 5 μm, UV = 210 nm), eluting with 60% MeOH in H_2_O to afford pratensilin D (**1**, 1.6 mg), tetrangulol (**4**, 4.1 mg), and 8-*O*-methyltetrangulol (**5**, 3.6 mg). The CH_2_Cl_2_/MeOH 49:1 fraction was purified by the same preparative HPLC system eluting with 45% MeOH in H_2_O to give kiamycin E (**2**, 1.2 mg) and pratensinon A (**3**, 0.6 mg). Chiral resolution of **1** was performed on Agilent analytical HPLC system [(*R, R*) WHELK-01 column, 4.6 mm × 250 mm, 10 μm, 100 A, iso-Pro-OH /n-Hexane = 20:80, 1.0 ml/min, UV = 210 nm] to obtain optically pure (+)-**1** (0.6 mg) and (–)-**1** (0.6 mg).

(+)-Pratensilin D [(+)-**1**]: colorless solid; ${[\alpha ]}_{\text{D}}^{25}$ + 50.5 (MeOH, *c* 0.2); UV (acetonitrile) λ_max_ (log ε) 211 (3.8), 241 (3.5), 302 (2.8), 343 (2.3) nm; ^1^H-NMR (600 MHz, DMSO-*d*_6_) and ^13^C-NMR (150 MHz, DMSO-*d*_6_) data, see Table S1; HRESIMS [M + H]^+^, *m/z* 468.14418 (calcd for C_28_H_22_O_6_N, 468.14471).

(–)-Pratensilin D [(–)-**1**]: colorless solid; ${[\alpha ]}_{\text{D}}^{25}$ – 61.8 (MeOH, *c* 0.2); UV (acetonitrile) λ_max_ (log ε) 211 (3.9), 241 (3.5), 302 (2.9), 345 (2.4) nm; ^1^H-NMR (600 MHz, DMSO-*d*_6_) and ^13^C-NMR (150 MHz, DMSO-*d*_6_) data, see Table S1; HRESIMS [M + H]^+^, *m/z* 468.14418 (calcd for C_28_H_22_O_6_N, 468.14471).

Kiamycin E (**2**): colorless solid; ${[\alpha ]}_{\text{D}}^{25}$ + 38.7 (MeOH, *c* 0.2); UV (acetonitrile) λ_max_ (log ε) 203 (3.6), 262 (2.8), 336 (2.5) nm; ^1^H-NMR (600 MHz, DMSO-*d*_6_) and ^13^C-NMR (150 MHz, DMSO-*d*_6_) data, see Table S2; HRESIMS [M + H]^+^, *m/z* 325.10740 (calcd for C_19_H_17_O_5_, 325.10760).

Pratensinon A (**3**): colorless solid; UV (acetonitrile) λ_max_ (log ε) 190 (3.2), 215 (3.09), 257 (3.12), 282 (2.6) nm; ^1^H-NMR (600 MHz, CDCl_3_) and ^13^C-NMR (150 MHz, CDCl_3_) data, see Table S3; HRESIMS [M + H]^+^, *m/z* 295.09677 (calcd for C_18_H_15_O_4_, 295.09703).

### X-Ray Crystallographic Analysis

Crystallographic data of **1**: X-ray quality crystals were acquired by slow volatilization of a solvent mixture of MeOH and CH_2_Cl_2_. Crystal data for xzp6: C_28_H_21_NO_6_, *M* = 467.46, *a* = 29.4142(7) Å, *b* = 12.1834(3) Å, *c* = 13.7172(3) Å, α = 90°, β = 114.8000(10)°, γ = 90°, *V* = 4462.42(18) Å^3^, T = 100. (2) K, space group *C*12/*c*1, *Z* = 8, μ(Cu Kα) = 0.812 mm^−1^, 41,600 reflections measured, 4,411 independent reflections (*R*_*int*_ = 0.0440). The final *R*_1_ values were 0.0419 [*I* > *2*σ(*I*)]. The final *wR*(*F*^2^) values were 0.1594 [*I* > *2*σ(*I*)]. The final *R*_1_ values were 0.0446 (all data). The final *wR*(*F*^2^) values were 0.1639 (all data). The goodness of fit on *F*^2^ was 1.450. Crystallographic data for compound **1** has been deposited in the Cambridge Crystallographic Data Centre with deposition number 1953675.

Crystallographic data of **2**: X-ray quality crystals were acquired by slow volatilization of a solvent mixture of MeOH and CH_2_Cl_2_. Crystal data for xzp8: C_19_H_16_O_5_, *M* = 324.32, *a* = 24.9006(5) Å, *b* = 8.1765(2) Å, *c* = 7.4089(2) Å, α = 90°, β = 100.7240(10)°, γ = 90°, *V* = 1482.11(6) Å^3^, *T* = 100. (2) K, space group *C*121, *Z* = 4, μ(Cu Kα) = 0.874 mm^−1^, 13,063 reflections measured, 2,919 independent reflections (*R*_*int*_ = 0.0315). The final *R*_1_ values were 0.0281 [*I* > 2σ(*I*)]. The final *wR*(*F*^2^) values were 0.0759 [*I* > 2σ(*I*)]. The final *R*_1_ values were 0.0287 (all data). The final *wR*(*F*^2^) values were 0.0766 (all data). The goodness of fit on *F*^2^ was 1.023. Flack parameter = 0.07(5). Crystallographic data for compound **2** has been deposited in the Cambridge Crystallographic Data Centre with deposition number 1992536.

### Bioactivity Assay

The antimicrobial assays of compounds **1**–**3** were tested against Gram-positive (*B. cereus* CMCC 32210, *S. aureus* CMCC 26003) bacteria and yeast (*C. albicans* CMCC 98001) using a microplate assay (Pierce et al., 2008). Nystatin and penicillin were used as positive controls against fungi and bacteria, respectively.

The NCI-H460 (non-small-cell lung carcinoma), PANC-1 (pancreatic cancer), HepG2 (liver hepatocellular carcinoma), Colon 38 (colon cancer), and Hela (cervical carcinoma) cells were plated at a density of 5,000 cells/well in 100 μl Dulbecco's modified Eagle's medium (DMEM). All cell lines were incubated overnight then treated with various concentrations of purified compounds in triplicate. After culturing for 72 h, 20 μl/well of 3-(4,5-dimethylthiazol-2-yl)-2,5-diphenyl tetrazolium bromide (MTT) solution (5 mg/ml, Sigma-Aldrich, USA) was added to each well, plate was cultured for 4 h at 37°C in a 5% CO_2_ atmosphere, which was followed by adding 150 μl DMSO to dissolve the formazan crystals, and shaking for 5 min. The absorbance was recorded at 570 nm by a microplate reader. IC_50_ value was taken using GraphPad Prism 5 software. Adriamycin was used as a positive control.

## Data Availability Statement

The datasets presented in this study can be found in online repositories. The names of the repository/repositories and accession number(s) can be found in the article/Supplementary Material.

## Author Contributions

LG, LZ, and QY separated and purified the compounds. ML and ZL identified the structures. BX and XF tested cytotoxic and antimicrobial activities. SZ and ZX conceived and designed the experiments. ZX prepared the paper. All authors approved the final manuscript. All authors contributed to the article and approved the submitted version.

## Conflict of Interest

The authors declare that the research was conducted in the absence of any commercial or financial relationships that could be construed as a potential conflict of interest.
