# Supplementary material for: Antibacterial and Cytotoxic Bridged and Ring Cleavage Angucyclinones From a Marine Streptomyces sp
Source: Front Chem. 2020 Aug 4;8:586. doi: 10.3389/fchem.2020.00586 (PMC7417440; doi:10.3389/fchem.2020.00586)
Supplement: Supplementary file 1 [file Table_1.DOCX]

**Supporting Information**

**Antibacterial and Cytotoxic Bridged and** **Ring Cleavage Angucyclinones from a Marine *Streptomyces* sp.**

Lin Guo^1^, Lu Zhang^1^, Qiaoli Yang^2^, Bo Qu^2^, Xinzhen Fu,^1^ Ming Liu^1^, Zhi Li^1^, Shumin Zhang^1^^*^ and Zeping Xie^1*^

^1^School of Pharmacy, Binzhou Medical University, Yantai, China

^2^College of Life Sciences, Yantai University, Yantai, China

*** Correspondence:**Zeping Xie
zepingxie@sina.com

Shumin Zhang

shumin_zhang@outlook.com

**Table of Contents**

[Table S1. NMR spectroscopic data for pratensilin D (**1**) in DMSO-*d_6_^a^* 4](#_Toc36113676)

[Table S2. NMR data for kiamycin E (**2**) in DMSO-*d_6_^a^* 6](#_Toc36113677)

[Table S3. NMR data for pratensinon A (**3**) in DMSO-*d_6_^a^* 8](#_Toc36113678)

[Figure S1. ^1^H NMR Spectrum (600 MHz) of (±)-Pratensilin D (**1**) in DMSO-*d_6_* 9](#_Toc36113679)

[Figure S2. ^13^C NMR Spectrum (150 MHz) of (±)-Pratensilin D (**1**) in DMSO-*d_6_* 10](#_Toc36113680)

[Figure S3. ^1^H-^1^H COSY Spectrum (600 MHz) of (±)-Pratensilin D (**1**) in DMSO-*d_6_* 11](#_Toc36113681)

[Figure S4. HSQC Spectrum (600 MHz) of (±)-Pratensilin D (**1**) in DMSO-*d_6_* 12](#_Toc36113681)

[Figure S5. HMBC Spectrum (600 MHz) of (±)-Pratensilin D (**1**) in DMSO-*d_6_* 13](#_Toc36113682)

[Figure S6. NOESY Spectrum (600 MHz) of (±)-Pratensilin D (**1**) in DMSO-*d_6_* 14](#_Toc36113682)

[Figure S7. ^1^H NMR Spectrum (600 MHz) of Kiamycin E (**2**) in DMSO-*d_6_* 15](#_Toc36113683)

[Figure S8. ^13^C NMR Spectrum (150 MHz) of Kiamycin E (**2**) in DMSO-*d_6_* 16](#_Toc36113684)

[Figure S9. DEPT-135 Spectrum (150 MHz) of Kiamycin E (**2**) in DMSO-*d_6_* 17](#_Toc36113685)

[Figure S10. ^1^H-^1^H COSY Spectrum (600 MHz) of Kiamycin E (**2**) in DMSO-*d_6_* 18](#_Toc36113686)

[Figure S11. HSQC Spectrum (600 MHz) of Kiamycin E (**2**) in DMSO-*d_6_* 19](#_Toc36113687)

[Figure S12. HMBC Spectrum (600 MHz) of Kiamycin E (**2**) in DMSO-*d_6_* 20](#_Toc36113688)

[Figure S13. NOESY Spectrum (600 MHz) of Kiamycin E (**2**) in DMSO-*d_6_* 21](#_Toc36113689)

[Figure S14. ^1^H NMR Spectrum (600 MHz) of Pratensinon A (**3**) in CDCl_3_ 22](#_Toc36113690)

[Figure S15. ^13^C NMR Spectrum (150 MHz) of Pratensinon A (**3**) in CDCl_3_ 23](#_Toc36113691)

[Figure S16. DEPT-135 Spectrum (150 MHz) of Pratensinon A (**3**) in CDCl_3_ 24](#_Toc36113692)

[Figure S17. ^1^H-^1^H COSY Spectrum (600 MHz) of Pratensinon A (**3**) in CDCl_3_ 25](#_Toc36113693)

[Figure S18. HSQC Spectrum (600 MHz) of Pratensinon A (**3**) in CDCl_3_ 26](#_Toc36113694)

[Figure S19. HMBC Spectrum (600 MHz) of Pratensinon A (**3**) in CDCl_3_ 27](#_Toc36113695)

[Figure S20. NOESY Spectrum (600 MHz) of Pratensinon A (**3**) in CDCl_3_ 28](#_Toc36113696)

[Figure S21. HRESIMS Spectrum of (±)-Pratensilin D (**1**). 29](#_Toc36113697)

[Figure S22. HRESIMS Spectrum of Kiamycin E (**2**). 30](#_Toc36113698)

[Figure S23. HRESIMS spectrum of Pratensinon A (3). 31](#_Toc36113699)

[Table S4. Crystal data and structure refinement for (±)-pratensilin D (**1**). 32](#_Toc36113700)

[Table S5. Crystal data and structure refinement for kiamycin E (**2**). 34](#_Toc36113701)

## Table S1. NMR spectroscopic data for pratensilin D (1) in DMSO-*d_6_^a^*

| position | *δ*_C_*^b^* | *δ*_H_ mult (*J* in Hz) | COSY | HMBC |
| --- | --- | --- | --- | --- |
| 1 |  | 156.4, C |  |  |
| 2 | 6.41, s | 103.7, CH | 3-CH_3_, 4 | 1, 3-CH_3_, 4, 12b |
| 3 |  | 136.0, C |  |  |
| 4 | 6.96, d (1.0) | 115.7, CH | 2, 3-CH_3_ | 1, 2, 3-CH_3_, 4a, 12b |
| 4a |  | 125.1, C |  |  |
| 5 | 7.54, d (8.6) | 127.8, CH | 6 | 1, 4, 6, 6a, 12b |
| 6 | 7.08, d (8.6) | 121.4, CH | 5 | 4a, 6a, 12, 12a |
| 6a |  | 149.3, C |  |  |
| 7 |  | 165.1, C |  |  |
| 7a |  | 116.5, C |  |  |
| 8 |  | 157.2, C |  |  |
| 9 | 7.25, d (7.9) | 114.0, CH | 8-OCH_3_, 10 | 7, 7a, 8, 11 |
| 10 | 7.55, t (7.9) | 135.6, CH | 9, 11 | 8, 9, 11a, 12 |
| 11 | 6.60, dd (7.9, 0.5) | 115.0, CH | 13 | 7a, 8, 9, 12 |
| 11a |  | 146.4, C |  |  |
| 12 |  | 104.6, C |  |  |
| 12a |  | 114.8, C |  |  |
| 12b |  | 128.2, C |  |  |
| 1’ |  | 133.8, C |  |  |
| 2’ | 7.15, dd (7.7, 1.4) | 131.2, CH | 3’ | 1’, 4’, 6’ |
| 3’ | 7.39, td (7.7, 1.6) | 132.5, CH | 2’, 4’ | 1’, 5’ |
| 4’ | 7.31, td (7.7, 1.4) | 129.1, CH | 3’, 5’ | 2’, 6’ |
| 5’ | 7.61, dd (7.7, 1.6) | 130.4, CH | 4’ | 1’, 3’, 7’ |
| 6’ |  | 132.9, C |  |  |
| 7’ |  | 166.4, C |  |  |
| 8’ | 3.75, s | 52.6, CH_3_ |  | 7’ |
| 3-CH_3_ | 2.35, s | 22.7, CH_3_ | 2, 4 | 2, 3, 4 |
| 8-OCH_3_ | 3.97, s | 56.3, CH_3_ | 9 | 8 |
| 6a-OH | 10.11, s |  |  |  |

*^a^*600 MHz for ^1^H NMR and 150 MHz for ^13^C NMR. *^b^*Numbers of attached protons were determined by analysis of 2D spectra.

## Table S2. NMR data for kiamycin E (2) in DMSO-*d_6_^a^*

| position | *δ*_H_ mult (*J*, Hz) | *δ*_C_*^b^* | HMBC | COSY | NOSEY |
| --- | --- | --- | --- | --- | --- |
| 1 |  | 154.3 C |  |  |  |
| 2 | 6.48, s | 113.4, CH | 1, 3-CH_3_, 4, 12a, 12b | 3-CH_3_ |  |
| 3 |  | 137.0, C |  |  |  |
| 4 | 6.45, s | 120.4, CH | 1, 2, 3-CH_3_, 4a, 5, 12b | 3-CH_3_, 5 |  |
| 4a |  | 135.9, C |  |  |  |
| 5 | 3.10, d (16.9)  2.82, dd (16.9, 2.8) | 35.3, CH_2_ | 1, 4, 4a, 6, 6a, 12, 12b | 4, 6 |  |
| 6 | 4.06, t (2.8) | 74.8, CH | 4a, 6a, 7, 12, 12a | 5 |  |
| 6a |  | 81.9, C |  |  |  |
| 7 |  | 205.8, C |  |  |  |
| 7a |  | 114.8, C |  |  |  |
| 8 |  | 162.2, C |  |  |  |
| 9 | 6.97, d (7.9) | 117.6, CH | 7a, 8, 10, 11 | 10 |  |
| 10 | 7.56, t (7.9) | 137.4, CH | 7a, 8, 11a | 9, 11 |  |
| 11 | 6.98, d (7.9) | 117.2, CH | 7a, 9, 11a, 12 | 10 |  |
| 11a |  | 148.8, C |  |  |  |
| 12 | 4.86, s | 85.0, CH | 6, 6a, 7a, 8, 11, 12a, 12b |  | 12a |
| 12a | 3.65, s | 49.0, CH | 1, 4a, 6, 6a, 7, 11a, 12, 12b |  | 6a-OH, 12 |
| 12b |  | 120.9, C |  |  |  |
| 3-CH_3_ | 2.18, s | 21.4, CH_3_ | 2, 3, 4 | 2, 4 |  |
| 1-OH | 9.30, s |  | 1, 2, 12b |  |  |
| 6a-OH | 6.08, s |  | 6, 6a, 12a |  | 12a |
| 8-OH | 11.69, s |  |  |  |  |

*^a^*600 MHz for ^1^H NMR and 150 MHz for ^13^C NMR. *^b^*Numbers of attached protons were determined by analysis of 2D spectra.

## Table S3. NMR data for pratensinon A (3) in DMSO-*d_6_^a^*

| position | *δ*_H_ mult (*J*, Hz) | *δ*_C_*^b^* | HMBC | COSY |
| --- | --- | --- | --- | --- |
| 1 |  | 205.7 C |  |  |
| 2 | 2.59, s | 31.1, CH_3_ | 1, 12b |  |
| 3 |  |  |  |  |
| 4 | 2.38, s | 19.0, CH_3_ | 4a, 5, 12b | 5, 6 |
| 4a |  | 139.1, C |  |  |
| 5 | 7.63, d (7.9) | 136.6, CH | 4, 6a, 12b | 4, 6 |
| 6 | 8.22, d (7.9) | 127.7, CH | 4a, 7, 12a | 4, 5 |
| 6a |  | 133.4, C |  |  |
| 7 |  | 181.8, C |  |  |
| 7a |  | 121.0, C |  |  |
| 8 |  | 160.4, C |  |  |
| 9 | 7.36, dd (8.5, 1.1) | 118.3, CH | 11 | 8-OCH_3_, 10 |
| 10 | 7.73, dd (8.5, 7.7) | 135.2, CH | 8, 11a | 9, 11 |
| 11 | 7.89, d (7.7, 1.1) | 120.1, CH | 7a, 9, 12 | 10 |
| 11a |  | 135.3, C |  |  |
| 12 |  | 183.8, C |  |  |
| 12a |  | 129.1, C |  |  |
| 12b |  | 142.6, C |  |  |
| 8-OCH_3_ | 4.06, s | 56.6, CH_3_ | 8 | 9 |

*^a^*600 MHz for ^1^H NMR and 150 MHz for ^13^C NMR. *^b^*Numbers of attached protons were determined by analysis of 2D spectra.

## Figure S1. ^1^H NMR Spectrum (600 MHz) of (±)-Pratensilin D (1) in DMSO-*d_6_*.

Figure S2. ^13^C NMR Spectrum (150 MHz) of **(±)-**Pratensilin D (1) in DMSO-*d_6_*.

Figure S3. ^1^H-^1^H COSY Spectrum (600 MHz) of (±)-Pratensilin D (1) in DMSO-*d_6_*.

Figure S4. HSQC Spectrum (600 MHz) of (±)-Pratensilin D (1) in DMSO-*d_6_*.

## Figure S5. HMBC Spectrum (600 MHz) of (±)-Pratensilin D (**1**) in DMSO-*d_6_*.

 Figure S6. NOESY Spectrum (600 MHz) of (±)-Pratensilin D (1) in DMSO-*d_6_*.

## Figure S7. ^1^H NMR Spectrum (600 MHz) of Kiamycin E (2) in DMSO-*d_6_*.

## Figure S8. ^13^C NMR Spectrum (150 MHz) of Kiamycin E (2) in DMSO-*d_6_*.

## Figure S9. DEPT-135 Spectrum (150 MHz) of Kiamycin E (2) in DMSO-*d_6_*.

## Figure S10. ^1^H-^1^H COSY Spectrum (600 MHz) of Kiamycin E (2) in DMSO-*d_6_*.

## Figure S11. HSQC Spectrum (600 MHz) of Kiamycin E (2) in DMSO-*d_6_*.

## Figure S12. HMBC Spectrum (600 MHz) of Kiamycin E (2) in DMSO-*d_6_*.

## Figure S13. NOESY Spectrum (600 MHz) of Kiamycin E (2) in DMSO-*d_6_*.

## Figure S14. ^1^H NMR Spectrum (600 MHz) of Pratensinon A (3) in CDCl_3_.

## Figure S15. ^13^C NMR Spectrum (150 MHz) of Pratensinon A (3) in CDCl_3_.

## Figure S16. DEPT-135 Spectrum (150 MHz) of Pratensinon A (3) in CDCl_3_.

## Figure S17. ^1^H-^1^H COSY Spectrum (600 MHz) of Pratensinon A (3) in CDCl_3_.

## Figure S18. HSQC Spectrum (600 MHz) of Pratensinon A (3) in CDCl_3_.

## Figure S19. HMBC Spectrum (600 MHz) of Pratensinon A (3) in CDCl_3_.

## Figure S20. NOESY Spectrum (600 MHz) of Pratensinon A (3) in CDCl_3_.

## Figure S21. HRESIMS Spectrum of (±)-Pratensilin D (1).


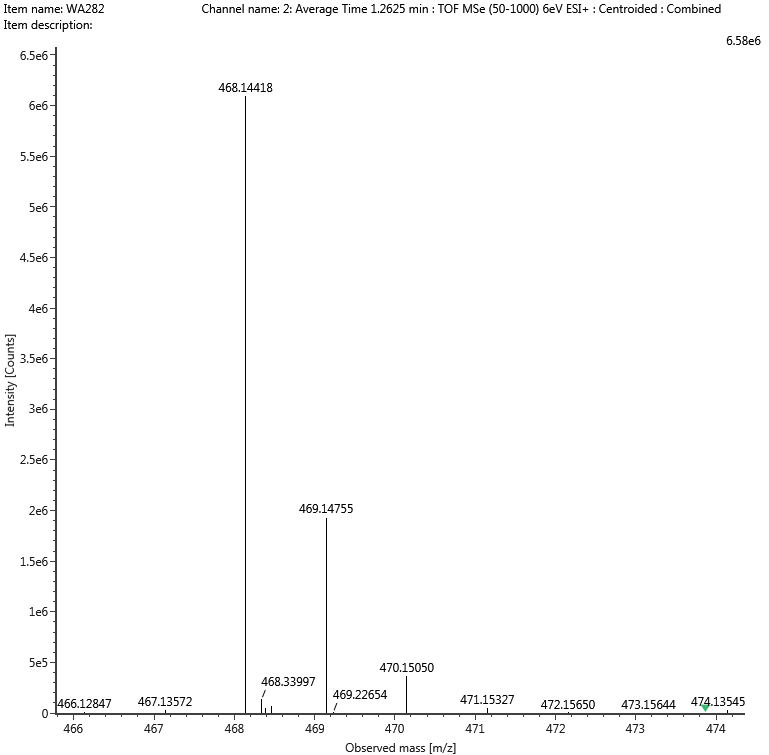


## Figure S22. HRESIMS Spectrum of Kiamycin E (2).


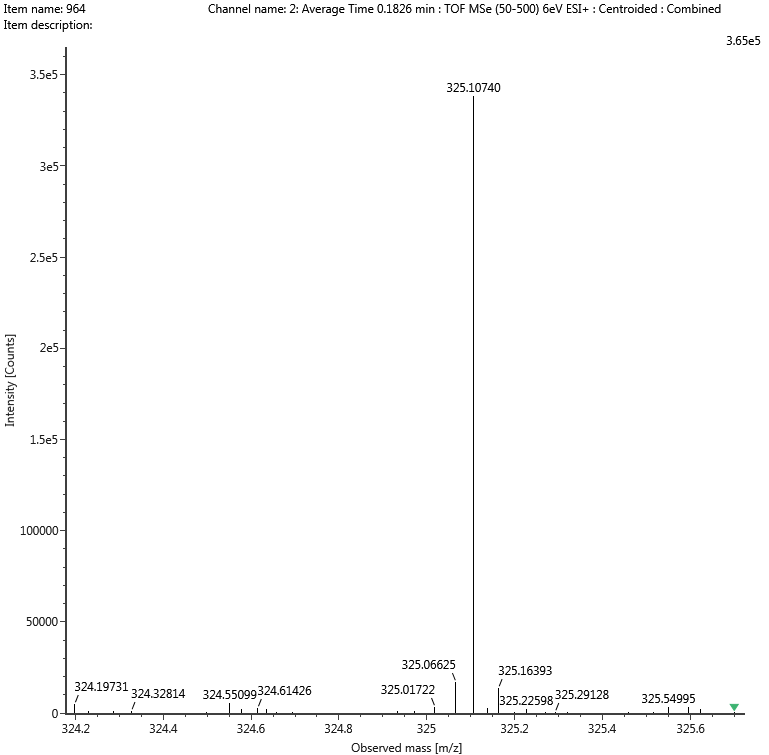


## Figure S23. HRESIMS Spectrum of Pratensinon A (3).


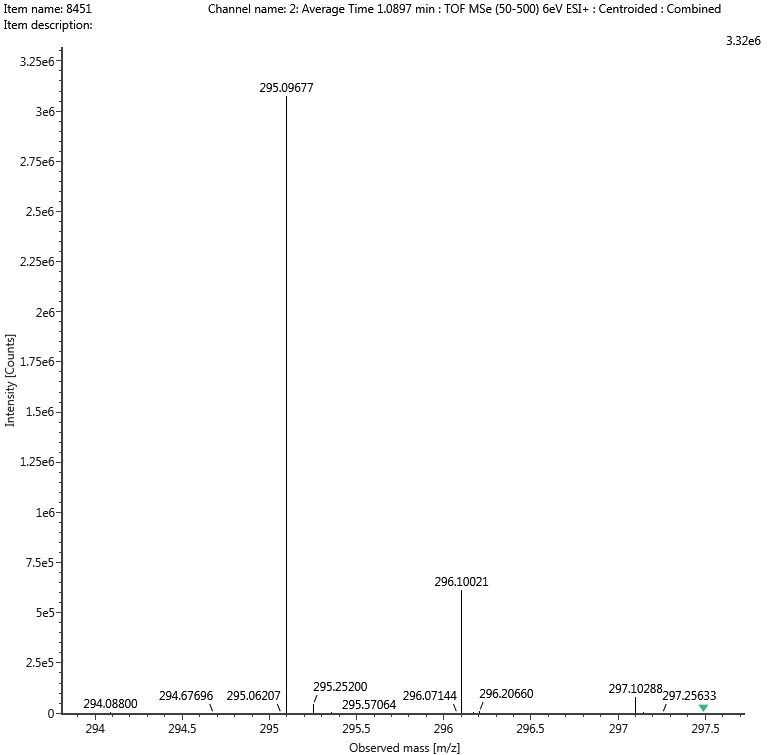


## **Table S4.** Crystal data and structure refinement for (±)-pratensilin D (**1**).

| Identification code | global |
| --- | --- |
| Empirical formula | C_28_H_21_NO_6_ |
| Formula weight | 467.46 |
| Temperature | 100(2) K |
| Wavelength | 1.54178 Å |
| Crystal system | Monoclinic |
| Space group | C 1 2/c 1 |
| Unit cell dimensions | a = 29.4142(7) Å α= 90°.  b = 12.1834(3) Å β= 114.8000(10)°.  c = 13.7172(3) Å γ = 90°. |
| Volume | 4462.42(18) Å3 |
| Z | 8 |
| Density (calculated) | 1.392 Mg/m3 |
| Absorption coefficient | 0.812 mm-1 |
| F(000) | 1952 |
| Crystal size | 0.440 x 0.220 x 0.110 mm3 |
| Theta range for data collection | 3.31 to 72.39°. |
| Index ranges | -36<=h<=36, -14<=k<=12, -16<=l<=16 |
| Reflections collected | 41600 |
| Independent reflections | 4411 [R(int) = 0.0440] |
| Completeness to theta = 72.39° | 99.7 % |
| Absorption correction | Semi-empirical from equivalents |
| Max. and min. transmission | 0.92 and 0.76 |
| Refinement method | Full-matrix least-squares on F2 |
| Data / restraints / parameters | 4411 / 0 / 321 |
| Goodness-of-fit on F2 | 1.450 |
| Final R indices [I>2sigma(I)] | R1 = 0.0419, wR2 = 0.1594 |
| R indices (all data) | R1 = 0.0446, wR2 = 0.1639 |
| Largest diff. peak and hole | 0.906 and -0.274 e.Å-3 |

## **Table S5.** Crystal data and structure refinement for kiamycin E (2).

| Identification code | global |
| --- | --- |
| Empirical formula | C_19_H_16_O_5_ |
| Formula weight | 324.32 |
| Temperature | 100(2) K |
| Wavelength | 1.54178 Å |
| Crystal system | Monoclinic |
| Space group | C 1 2 1 |
| Unit cell dimensions | a = 24.9006(5) Å α= 90°.  b = 8.1765(2) Å β= 100.7240(10)°.  c = 7.40892(2) Å γ = 90°. |
| Volume | 1482.11(6) Å3 |
| Z | 4 |
| Density (calculated) | 1.453 Mg/m3 |
| Absorption coefficient | 0.874 mm-1 |
| F(000) | 680 |
| Crystal size | 0.400 x 0.050 x 0.030 mm3 |
| Theta range for data collection | 3.61 to 72.36°. |
| Index ranges | -30<=h<=29, -10<=k<=10, -8<=l<=9 |
| Reflections collected | 13063 |
| Independent reflections | 2919 [R(int) = 0.0315] |
| Completeness to theta = 72.39° | 99.6 % |
| Absorption correction | Semi-empirical from equivalents |
| Max. and min. transmission | 0.97 and 0.86 |
| Refinement method | Full-matrix least-squares on F2 |
| Data / restraints / parameters | 2919 / 1 / 222 |
| Goodness-of-fit on F2 | 1.023 |
| Final R indices [I>2sigma(I)] | R1 = 0.0281, wR2 = 0.0759 |
| R indices (all data) | R1 = 0.0287, wR2 = 0.0766 |
| Absolute structure parameter | 0.07(5) |
| Largest diff. peak and hole | 0.226 and -0.179 e.Å-3 |
